# Effects of short-term spinal cord stimulation on patients with prolonged disorder of consciousness: A pilot study

**DOI:** 10.3389/fneur.2022.1026221

**Published:** 2022-10-14

**Authors:** Yutong Zhuang, Yi Yang, Long Xu, Xueling Chen, Xiaoli Geng, Jizong Zhao, Jianghong He

**Affiliations:** ^1^Department of Neurosurgery, The Second School of Clinical Medicine, Southern Medical University, Guangzhou, China; ^2^Department of Neurosurgery, Beijing Tiantan Hospital, Capital Medical University, Beijing, China; ^3^China National Clinical Research Center for Neurological Diseases, Beijing, China

**Keywords:** disorder of consciousness, minimally conscious state, short-term spinal cord stimulation, frequency, neuromodulation

## Abstract

**Background:**

Spinal cord stimulation (SCS) can improve the level of awareness of prolonged disorder of consciousness (pDOC), but its application is restricted due to damage of invasive operation. Short-term spinal cord stimulation (st-SCS) in a minimally invasive manner will better balance the benefits and risks.

**Objectives:**

This study focuses on the safety and efficacy of st-SCS for pDOC and reveals the modulation characteristics of different frequencies of SCS.

**Methods:**

31 patients received 2-week st-SCS treatment and 3-months follow-up. All patients were divided into two types of frequency treatment groups of 5 Hz and 70 Hz according to the postoperative electroencephalography (EEG) test. The efficacy was assessed based on the revised coma recovery scale (CRS-R).

**Results:**

The results showed a significant increase in CRS-R scores after treatment (Z = −3.668, *p* < 0.001) without significant adverse effects. Univariate analysis showed that the minimally conscious state minus (MCS–) benefits most from treatment. Furthermore, two frequency have a difference in the time-point of the CRS-R score increase. 5 Hz mainly showed a significant increase in CRS-R score at 2 weeks of treatment (*p* = 0.027), and 70 Hz additionally showed a delayed effect of a continued significant increase at 1 week after treatment (*p* = 0.004).

**Conclusion:**

st-SCS was safe and effective in improving patients with pDOC levels of consciousness, and was most effective for MCS–. Both 5 Hz and 70 Hz st-SCS can promote consciousness recovery, with 70 Hz showing a delayed effect in particular.

## Introduction

pDOC refer to the state of awakening and not recovering consciousness for more than 28 days after severe brain injury, which is mainly classified into two diagnoses: vegetative state or unresponsive wakefulness syndrome (VS/UWS) and minimally conscious state (MCS). The former is characterized by the presence of a sleep-wake cycle but lack of consciousness, while the latter is characterized by the presence of fluctuating and reproducible signs of consciousness (1). In 2011, Bruno et al. identified heterogeneity in the MCS and further divided it into minimally conscious state minus (MCS–) and minimally conscious state plus (MCS+), with the former having signs of low-level consciousness responses and the latter with language-related cognitive abilities (2).

In the treatment field of pDOC, non-invasive neuromodulation such as transcranial direct current stimulation (tDCS) and repetitive transcranial magnetic stimulation (rTMS) have been widely used in clinical practice for their safety, simplicity, and non-invasiveness (3). In recent years, the mesocircuit model has suggested that loss of consciousness after severe brain injury may be due to disruption of cortico-thalamic and cortico-cortical connections (4). The principle of treatment of the non-invasive neuromodulation determines its scope of effect to modulate only the cortico-cortical connections. Deep Brain Stimulation (DBS) (5), spinal cord stimulation (SCS) (6), and vagus nerve stimulation (VNS) (7), can directly modulate the neural circuit and are expected to be an effective means to solve “disorders of consciousness (DOC).” DBS has been found to be an modulation for the thalamocortical and thalamostriate loops (8–10), but indications of DBS for DOC includes no significant lesions of thalamus and displacement of deep nuclear cluster to ensure accurate implantation of electrodes. Therefore, the strict indications make it impossible to perform in many pDOC.

SCS has become an important and valid surgical therapy for DOC because its operation procedure is relatively easy, safe, and has a wide range of indications. Kanno et al. (11) first proposed the application of SCS to pDOC and achieved promising results. Subsequently, DellaPepa et al. summarized multiple SCS studies and found that 51.6% patients with pDOC showed recovery of consciousness and inferred the treatment effect is that SCS activates the thalamocortical pathway and increases cerebral blood flow through the ascending reticular activating system (12). Our research team also reported that 31.8% patients showed improvement in consciousness (6), and the above findings suggest that SCS can effectively promote the recovery of consciousness. The overall effective rate of SCS ranges from 20 to 40% (13).

A study on factors influencing the efficacy of SCS found that pDOC patients with a short duration of disease had a better chance of recovery of consciousness (14). Yamamoto et al. have the same findings. All 10 pDOC patients who recovered consciousness underwent the operation of SCS within 9 months after brain injury (13). However, the disadvantages of SCS, such as the significant injuries caused by invasive operations and the potential risk of implant rejection, prevented its early application in DOC like TMS and tDCS. Therefore, SCS is usually used to treat pDOC patients with a duration of disease of more than 3 months to avoid the spontaneous high-speed recovery of consciousness (15). But, excessive waiting time may result in missing the golden window to receive treatment.

More broadly, the treatment of spinal cord stimulation includes SCS and st-SCS whose electrodes are placed percutaneously to the spinal epidural for 2 weeks. st-SCS was firstly used clinically to ease pain (16, 17), and it also used as experimental treatment to test for response of patients with pain to SCS. If there is significant analgesia, electrodes of SCS will be permanently implanted a few weeks later to maintain control of pain symptoms (18). It is now accepted that early stage pain patients are particularly suitable for this therapy (19).

According to clinical experience, we have found that different frequencies of SCS have caused various effects on different patients with pDOC. However, immediate behavioral change after single stimulation of SCS is hard to detect at bedside, which makes it difficult to adjust parameters after operation. Recently, different frequency activities of EEG have been found to play an important role in the assessment of intervention efficacy (20), of which enhanced delta activity and down-regulated alpha activity are now generally considered to be consistent markers of low levels of consciousness (21). A previous study by our team found that the relative power in the delta band was significantly lower in pDOC patients with single stimulation of SCS at 5 and 70 Hz compared to pre-stimulation (22).

Given the minimally invasive, simple, and low-risk advantage and the proven experience in the application of pain. We attempted to treat pDOC patients with st-SCS, aiming to minimize the injuries caused by operation, expand the beneficiary population of SCS and advance the time of intervention as much as possible, balancing to some extent the contradiction between the earlier time of spinal cord stimulation intervention and spontaneous high-speed recovery in the first three months of onset. Meanwhile, to exclude the possibility of unsuitable frequency for individuals leading to ineffective st-SCS treatment and reveal the characteristics of clinical modulation of different frequencies of SCS, the present study has two different frequencies treatment groups and individualized treatment frequency of st-SCS is selects by EEG.

## Materials and methods

### Study subjects

**Figure 1:**
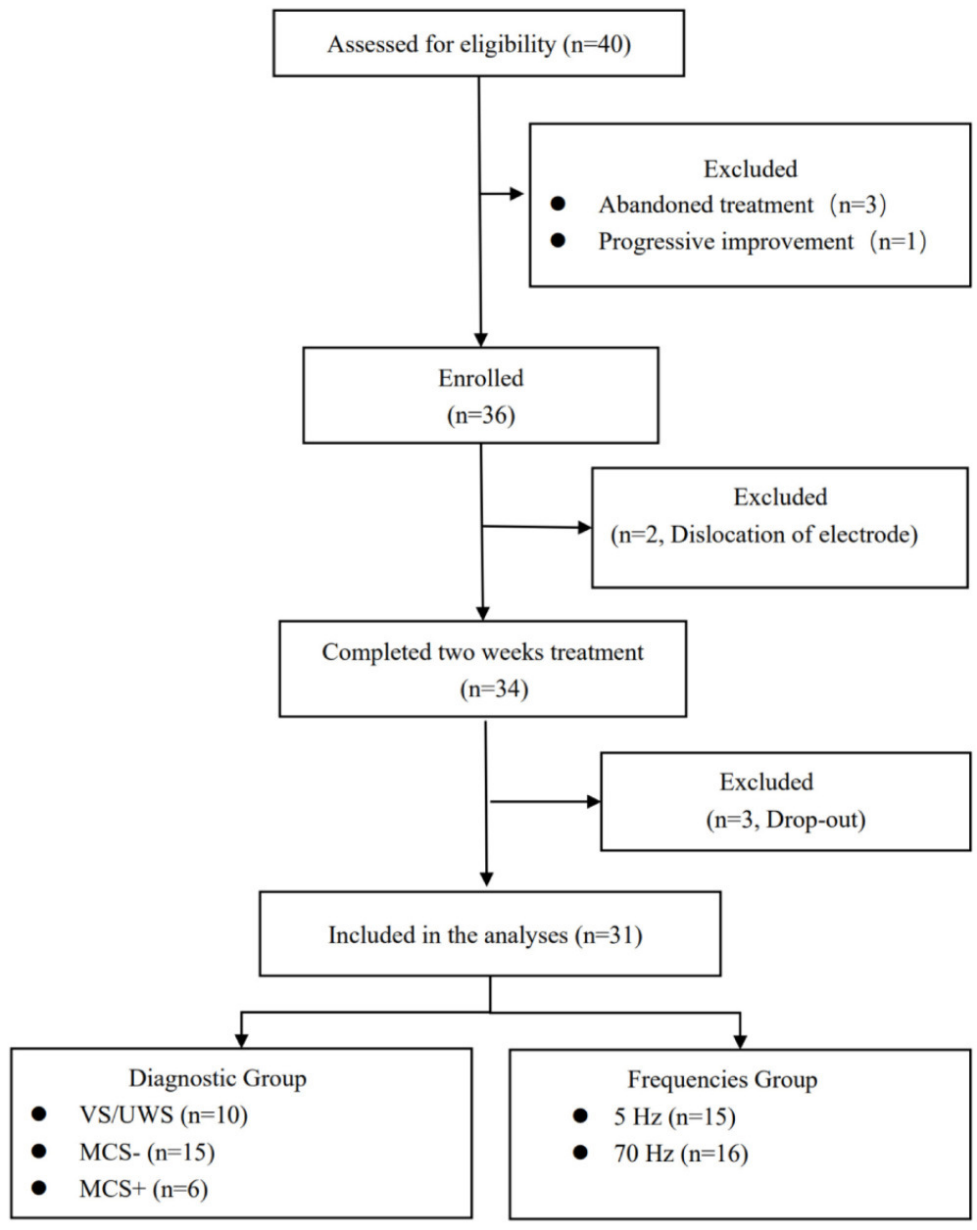
Study flow diagram. Diagnosis VS/UWS, vegetative state or unresponsive Wakefulness Syndrome; MCS−, minimally conscious state minus; MCS+, Minimally Conscious State plus.

Forty patients with pDOC were recruited for this study at Beijing Tiantan Hospital, Capital Medical University, from November 2021 to March 2022. 9 patients finally were excluded and the details is showed in Figure 1. 31 included patients were aged 18–67 years (45.19 ± 15.33), with the duration of disease of 3–23 months (7.78 ± 5.49), preoperative CRS-R score of 3–15 (8.52 ± 3.05), and gender (25 male/6 female). Their etiologies were 10 traumatic brain injury (TBI), 18 stroke, and 3 ischemia and anoxia (IAA). They were divided into three clinical diagnostic subgroups according to the CRS-R scale, including 10 VS/UWS, 15 MCS–, and 6 MCS+ (Table 1).

**Table 1:** Demographic details for patients.

**Patient**	**Gender**	**Age**	**Etiology**	**Post-injure (months)**	**CRS-R**						
					**Total**	**A**	**V**	**M**	**OM**	**C**	**Ar**
**VS/UWS**											
P1	F	53	S	18	5	1	0	1	1	0	2
P2	M	36	A	3	7	1	1	2	1	0	2
P3	F	32	S	4	7	1	1	2	1	0	2
P4	M	55	S	3	3	0	0	2	1	0	0
P5	F	67	T	5	6	1	1	2	1	0	1
P6	M	54	T	12	7	1	1	2	1	0	2
P7	F	20	S	13	4	1	0	1	1	0	1
P8	F	64	T	7	4	1	0	1	1	0	1
P9	F	59	S	5	7	1	1	2	1	0	2
P10	F	30	S	12	7	1	1	2	1	0	2
**MCS–**											
P1	F	63	T	6	11	2	3	3	1	0	2
P2	F	59	S	14	9	1	3	2	1	0	2
P3	F	48	S	23	10	1	0	5	2	0	2
P4	F	45	T	7	6	0	3	1	0	0	2
P5	F	21	T	5	8	0	3	2	1	0	2
P6	F	52	S	4	9	1	3	2	1	0	2
P7	F	18	A	6	8	1	3	1	1	0	2
P8	M	49	S	9	8	0	3	2	1	0	2
P9	F	64	S	8	8	1	3	1	1	0	2
P10	F	18	T	3	8	0	3	2	1	0	2
P11	F	61	T	5	11	0	3	1	1	0	2
P12	F	58	S	3	7	0	3	1	1	0	2
P13	F	56	S	4	8	1	3	2	1	0	2
P14	F	35	T	3	8	1	1	2	1	1	2
P15	F	34	H	4	8	1	1	3	1	0	2
**MCS+**											
P1	F	28	S	8	14	3	4	4	1	0	2
P2	M	31	T	9	15	3	4	5	1	0	2
P3	M	56	S	9	15	3	4	5	1	0	2
P4	F	40	A	5	12	3	4	3	0	0	2
P5	F	36	S	23	11	3	3	1	2	0	2
P6	F	59	S	4	13	3	3	2	2	1	2

Gender (F, female; M, male); Etiology (A, anoxic; T, traumatic brain injury; S, stroke); CRS-R, Coma recovery scale-revised (A, auditory function; V, visual; M, motor; OM, oromotor; C, communication; Ar, arousal).

All enrolled patients met the following inclusion criteria: (1) definitive diagnosis as DOC; (2) age 18–70 years; (3) duration of disease more than 3 months; (4) consciousness was in a stable phase for at least 4 weeks before enrollment and (5) patient's family members agreed to undergo the operation of st-SCS and had signed an informed consent form. Exclusion criteria included (1) neurodegenerative diseases such as Alzheimer's disease and Lewy body dementia; (2) coma caused or complicated by the deterioration of systemic diseases, or those who were not expected to survive long; (3) seizures that were difficult to control; (4) normal spine and spinal canal structure, no history of spinal cord injury, no cervical cone fracture or significant spinal stenosis, or other contraindications to operation; (5) those who are undergoing experimental drug or instrumentation trials.

### Surgical procedure

**Figure 2:**
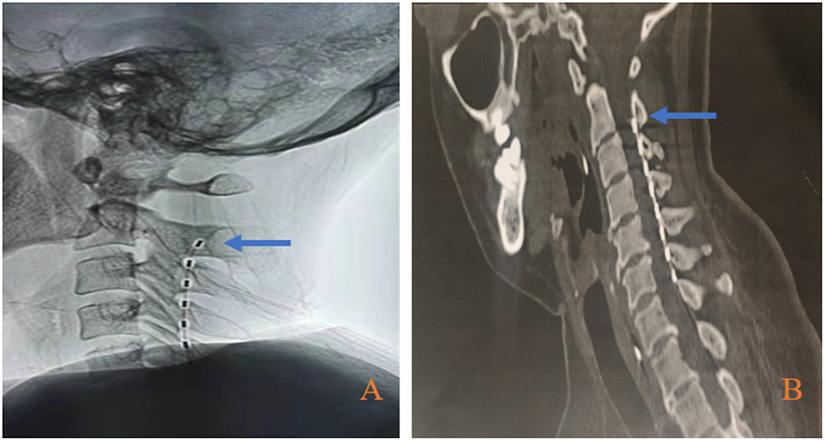
Electrode placement position. **(A)** Electrode position during operation; **(B)** Electrode position before electrode extraction. Bule arrow indicate the second cervical vertebra (C2) level.

**Figure 3:**
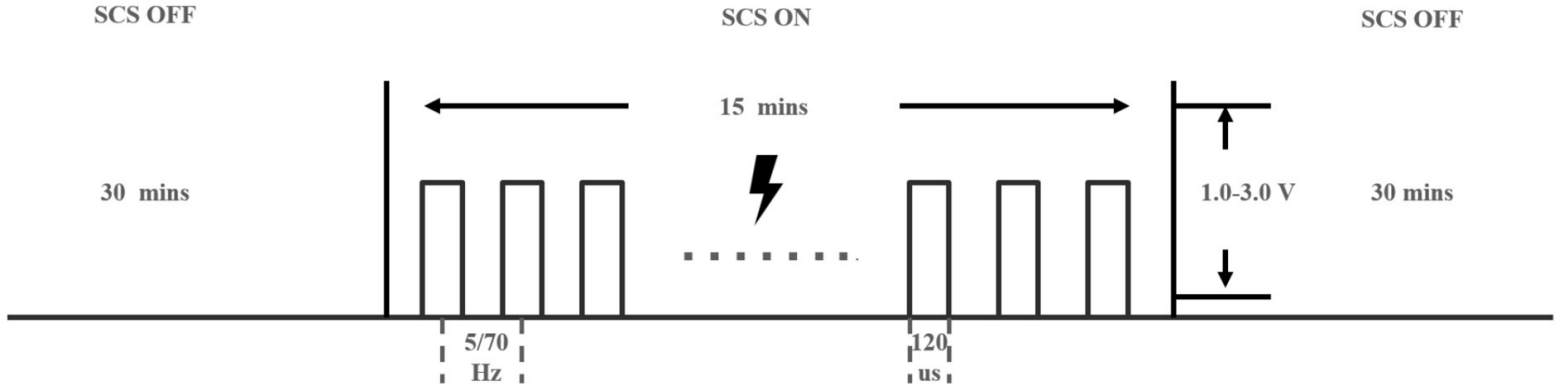
The stimulation paradigm of st-SCS.

The st-SCS operation is performed under general anesthesia as follows: (1) cervical MRI was performed before the operation to locate the target segment and spinal cord condition; (2) intraoperatively, the patient was placed in a prone position, with the neck flexed forward, and 8 contacts stimulation electrode (3777; Medtronic, Minneapolis, USA) was placed into the epidural space at the T7/8 level by skin puncture, and the tip of the electrode was implanted along the midline of the spinal cord to the C2 level under X-ray fluoroscopic guidance within the epidural space gap (Figure 2A); (3) the electrode extension was connected to an external pulse generator and battery (37022; Medtronic, Minneapolis, USA); (4) the puncture needle was withdrawn and the electrode leads were sutured and secured to the dorsal skin.

Cervical CT was reexamined within 24 h after the operation to observe the electrode position. The electrode was removed 2 weeks after the stimulation was turned on, and the cervical CT was reexamined within 24 h before the electrode removal to reconfirm the electrode position (Figure 2B) to ensure that this treatment process is an effective stimulation.

### EEG recording

EEG signals were recorded online at the bedside at a sampling rate of 500 Hz using an EEG acquisition device (Nicolet EEG V32, Natus Neurology, USA) with 32 Ag/AgCl electrodes based on the international standard 10–20 system setup. All electrodes were set with FCz as the reference electrode and AFz as the ground electrode. The impedance between the electrodes and the patient's skin was always kept below 5 kΩ. Patients were kept awake during EEG monitoring. 19 electrodes (Fp1, Fp2, F3, Fz, F4, F7, F8, Cz, C3, C4, Pz, P3, P4, O1, O2, T3, T4, T5, T6) were selected for off-line visual EEG, and the EEG display parameters were set to trap 50 Hz, band-pass filtered to 1–40 Hz, reference to average reference, sensitivity to 70 uV/cm, and paper walking speed to 30 mm/s.

### Stimulation protocol

The uppermost contact of the st-SCS was used for the stimulation contact cathode (0–1+2+). The stimulation pulse width was set to 120 us, the stimulation intensity ranged from 1.0 to 3.0 V, and the individualization stimulation intensity was set according to the Previous clinical study of SCS in the treatment of pDOC: 5 Hz stimulation induces bilateral upper limb tremors (13), and 70 Hz stimulation just did not induce significant limb movements (6).

Our prior study showed that frequency selection is crucial for the efficacy of spinal cord electrical stimulation (22). Therefore, in this study, patients were individually selected for appropriate frequencies based on the postoperative EEG test. The EEG test proceeded as follows: all patients received continuous stimulation at a single frequency of 5 or 70 Hz for 15 mins, and resting EEG was monitored for 30 mins before and after stimulation. The two frequency tests were at least 24 h apart to elute the residual effect of the last stimulus, and their sequences were performed in a pseudo-randomized manner. The test was completed 2 days after the operation (see Figure 3).

**Figure 4:**
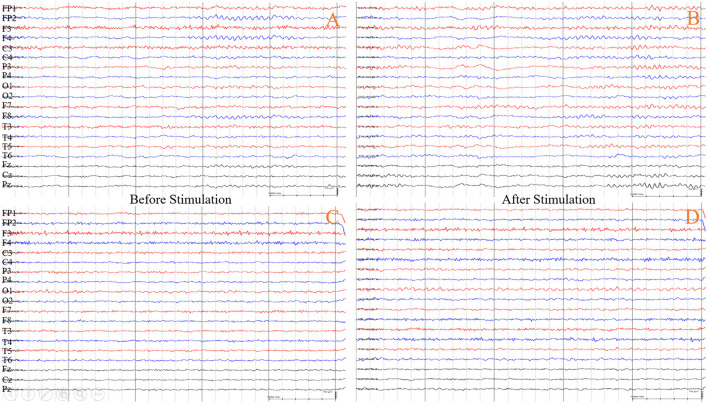
EEG responsiveness to different stimulation frequencies of st-SCS in a patient (he chose 5 Hz as the treatment frequency). **(A)** Before the stimulation at 5 Hz, the waking EEG background activity showed all lead is characterized by low-medium wave amplitude 9–10 Hz alpha rhythm activity, no obvious dominant rhythm in the occipital region, and poor modulation amplitude. **(B)** After stimulation at 5 Hz, the waking EEG background activity shows an increase in frequency to 11–12 Hz compared to the **(A)**, with improved modulation and higher amplitude. **(C,D)** Before and after stimulation at 70 Hz, the waking EEG background activity did not change significantly.

Two experienced electrophysiologists were offline and each independently visually observed changes in EEG background activity before and after stimulation, both without knowledge of the entire study. An increase in alpha rhythm (8–13 Hz) or a decrease in delta rhythm (1–4 Hz) was taken as an improvement in EEG activity. The treatment frequency was eventually set to the frequency that caused the best improvement in EEG after stimulation (Figure 4).

On-stimulation time was less than the off-stimulation time to reduce neuronal fatigue or damage (15). Therefore, the stimulation cycle was chosen to be 5 min ON/15 min OFF. To meet the patients' normal sleep requirements, stimulation was turned on at 8 am and off at 8 pm for a total of 2 weeks of on-stimulation treatment.

All patients underwent routine rehabilitation: passive limb training and swallowing function training throughout the study. In order to attribute the efficacy to stSCS as much as possible, the enrolled patients not underwent non-invasive neuromodulation treatments such as TMS and tDCS.

### Clinical assessments and follow-up

**Figure 5:**
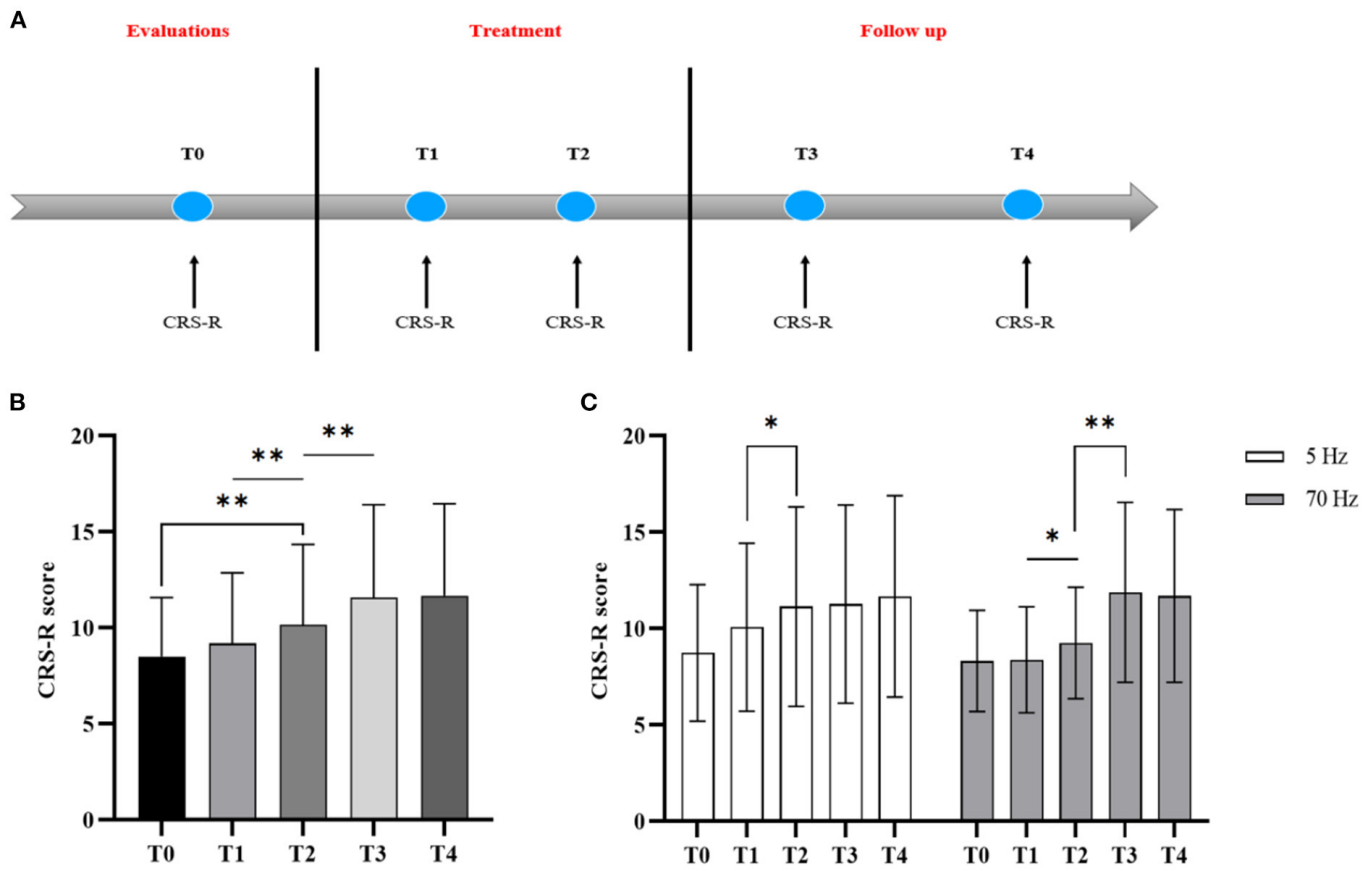
CRS-R changes with treatment going at five-time points: before the treatment (T0), treatment of 1 week (T1), treatment of 2 weeks (T2), 1 week after treatment (T3), and 3 months follow-up (T4). **(A)** Study protocol timeline showing treatment effects of stSCS evaluated with CRS-R, **(B)** CRS-R changes at five-time points, **(C)** CRS-R changes of different frequency groups at five-time points. Asterisk indicates significant differences based on One-way RMANOVA (*p < 0.05; **p < 0.01).

Changes in the patient's state of consciousness were assessed based on the CRS-R scale (1) in three phases: before treatment (2 weeks before operation, T0), treatment (1 week, T1, 2 weeks, T2), and post-treatment follow-up (1 week, T3, 3 months, T4) (Figure 5A). At least three times assessments by CRS-R were performed 2 weeks before the operation to clarify the patient's level of consciousness and clinical diagnosis before treatment. Effective clinical outcomes of st-SCS is that patients showed a clinical diagnostic improvement.

### Statistical analysis

IBM SPSS Statistics 26 software was used for statistical analysis. The effect of the time factor on the CRS-R score was analyzed by one-way repeated measures ANOVA (RMANOVA), and the effect of the frequency grouping factor and the time factor was analyzed by two-way RMANOVA. The *post hoc* test was adopted as the Least significant difference t-test; The difference in CRS-R scores before and after treatment was tested by Wilcoxon signed-rank test. And the difference between groups was tested by Mann-Whitney test for measurement data and were tested by the chi-square test or Fisher exact test for count data. The rate of change of subscale between pre-treatment and post-treatment [(mean post-treatment CRS-R – mean pre-treatment CRS-R)/(mean pre-treatment CRS-R + mean post-treatment CRS-R)] (23).

## Results

### Feasibility and safety

Fifiteen patients with pDOC of whom received 5 Hz stimulation and 16 of whom received 70 Hz stimulation. There were no epidural hematoma formation during electrode placement, and no seizures or intracranial infections during stimulation.

### Clinical effect of st-SCS treatment

Diagnostic improvement was found in 15 patients at 3 months of follow-up with an overall effective rate of 48% (15/31) (Table 2).

**Table 2:** Changes in CRS-R at different time-points.

**Patient**	**T0**	**T1**	**T2**	**T3**	**T4**	**Changes of diagnosis**
	**CRS-R: Total (A V M OM C Ar)**					
**VS/UWS**	
P1^b^	5	5	6	6	5	Remained VS/UWS
	(101102)	(101102)	(102102)	(102102)	(101102)	
P2^a^	7	5	5	5	4	Remained VS/UWS
	(112102)	(002102)	(002102)	(002102)	(002002)	
P3^b^	7	7	9	11	11	VS/UWS improved to MCS +
	(112102)	(112102)	(132102)	(332102)	(332102)	
P4^a^	3	4	4	4	4	Remained VS/UWS
	(002100)	(102100)	(102100)	(102100)	(102100)	
P5^b^	6	4	5	6	6	Remained VS/UWS
	(112101)	(102100)	(012101)	(012102)	(012102)	
P6^b^	7	7	7	7	7	Remained VS/UWS
	(112102)	(112102)	(112102)	(112102)	(112102)	
P7^a^	4	4	4	4	4	Remained VS/UWS
	(100102)	(100102)	(100102)	(100102)	(100102)	
P8^b^	4	5	6	8	8	VS/UWS improved to MCS +
	(101101)	(101102)	(111102)	(311102)	(311102)	
P9^a^	7	7	7	7	7	Remained VS/UWS
	(112102)	(112102)	(112102)	(112102)	(112102)	
P10^b^	7	7	7	7	8	Remained VS/UWS
	(112102)	(112102)	(112102)	(112102)	(112202)	
**MCS–**						
P1^a^	11	18	18	18	14	MCS– improved to MCS+
	(233102)	(346113)	(346113)	(346113)	(244112)	
P2^b^	9	9	9	18	15	MCS– improved to MCS+
	(123102)	(123102)	(123102)	(456102)	(345102)	
P3^b^	10	11	12	14	14	MCS– improved to MCS+
	(105202)	(105302)	(105312)	(305312)	(305312)	
P4^a^	6	8	9	11	13	Remained MCS–
	(031002)	(032102)	(132102)	(133202)	(133202)	
P5^b^	8	9	9	18	18	MCS– improved to EMCS
	(032102)	(132102)	(132102)	(453123)	(453123)	
P6^b^	9	8	11	16	16	MCS– improved to EMCS
	(132102)	(032102)	(332102)	(452122)	(452122)	
P7^a^	8	13	15	15	18	MCS– improved to MCS+
	(131102)	(343102)	(345102)	(345102)	(455112)	
P8^b^	8	8	8	8	8	Remained MCS–
	(032102)	(032102)	(032102)	(032102)	(032102)	
P9^b^	8	8	8	11	11	MCS– improved to MCS+
	(132101)	(132101)	(132101)	(332102)	(332102)	
P10^b^	8	9	14	20	20	MCS– improved to EMCS
	(032102)	(132102)	(333302)	(453323)	(453323)	
P11^b^	11	11	11	14	14	MCS– improved to MCS+
	(233102)	(233102)	(233102)	(343202)	(343202)	
P12^a^	7	9	10	10	10	Remained MCS–
	(031102)	(231102)	(232102)	(232102)	(232102)	
P13^a^	8	10	10	10	16	MCS– improved to MCS+
	(113102)	(313102)	(313102)	(313102)	(315322)	
P14^a^	8	15	17	17	17	MCS– improved to MCS+
	(112112)	(345102)	(453112)	(453112)	(453112)	
P15^a^	8	8	11	11	11	MCS– improved to MCS+
	(113102)	(113102)	(313112)	(313112)	(313112)	
**MCS+**						
P1^a^	14	14	14	14	14	Remained MCS+
	(344102)	(344102)	(344102)	(344102)	(344102)	
P2^a^	15	15	21	21	20	MCS+ Improved to EMCS
	(345102)	(345102)	(456123)	(456123)	(456122)	
P3^b^	15	15	15	15	15	Remained MCS+
	(345102)	(345102)	(345102)	(345102)	(345102)	
P4^a^	12	8	9	9	9	Remained MCS+
	(343002)	(231002)	(331002)	(331002)	(331002)	
P5^b^	11	11	11	11	11	Remained MCS+
	(331202)	(331202)	(331202)	(331202)	(331202)	
P6^a^	13	13	13	13	14	Remained MCS+
	(332212)	(332212)	(332212)	(332212)	(332213)	

Clinical diagnosis (VS/UWS, vegetative state or unresponsive wakefulness syndrome; MCS−, minimally conscious state minus; MCS+, minimally conscious state plus; EMCS, emerged from MCS); Frequencies (^a^5 Hz; ^b^70 Hz); CRS-R, Coma recovery scale-revised (A, auditory function; V, visual; M, motor; OM, oromotor; C, communication; Ar, arousal).

**Figure 6:**
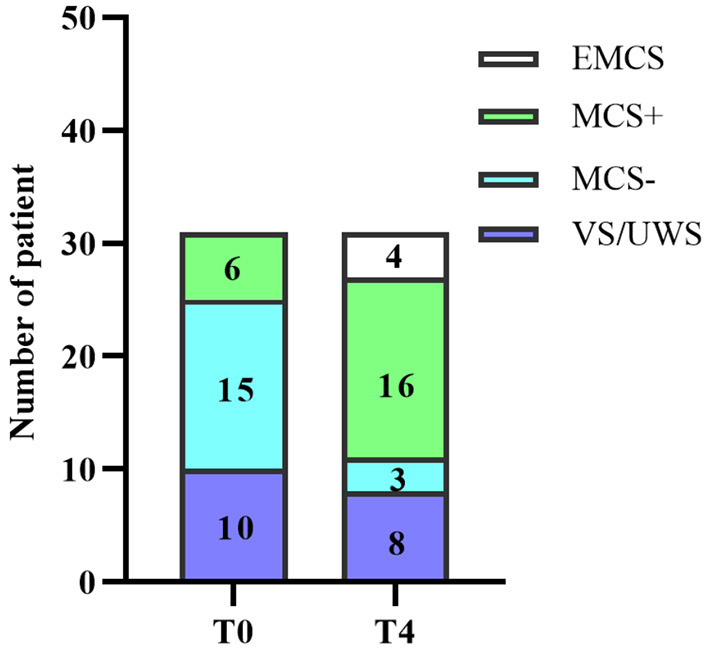
Changes in clinical diagnosis before and after treatment. T0: before the treatment; T4: 3 months follow-up.

**Figure 7:**
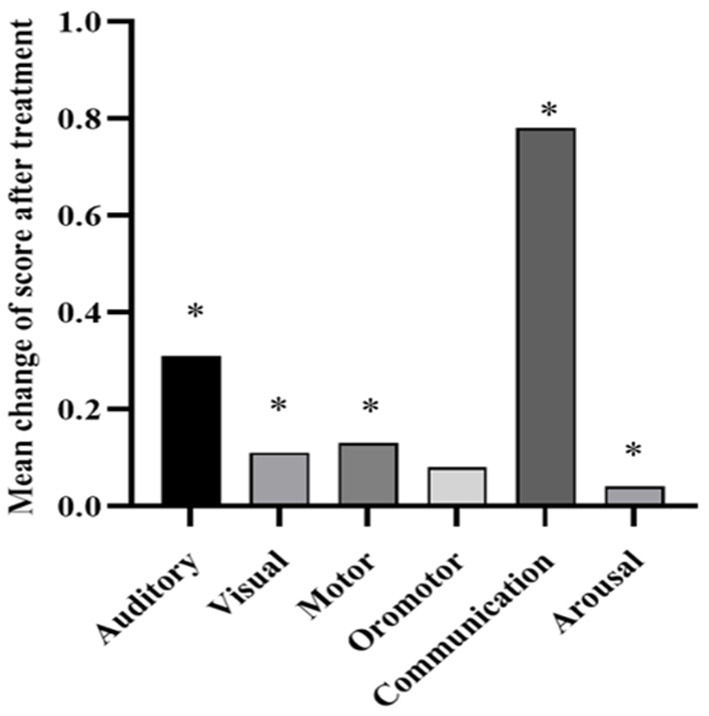
CRS-R subscale change rate before and after treatment. Wilcoxon signed-rank test indicates significant differences (*p < 0.05).

The MCS had an effective rate of 62% (13/21), while the VS/UWS is 20% (2/10), however, there was no significant difference in effectiveness between the two diagnostic groups (2 × 2 Fisher exact test, *p* = 0.054). Further subdivision of the MCS diagnostic revealed a statistical difference between the effective and ineffective groups for the three diagnostic subgroups (2 × 3 Fisher exact test, *p* = 0.002) (Table 3). The MCS– had an effective rate of 80% (12/15) and the MCS+ is 17% (1/6). Further *post hoc* revealed that st-SCS for MCS– had significantly higher effective rate than VS/UWS (OR = 16, 95% CI: 2.165–118.27; *p* = 0.005) and MCS+ (OR = 20, 95% CI: 1.655–241.723; *p* = 0.014), while there was no statistically significant difference between MCS+ and VS/UWS with similar effective rate (*p* > 0.05). Specifically, 20% VS/UWS improved to MCS+, 75% MCS– improved to MCS+ But, only 25% MCS– improved to EMCS, and similarly only 17% MCS+ improved to EMCS (Figure 6). As for the CRS-R subscale (Figure 7), except for arousal function (Z = −1.613, *p* = 0.107), st-SCS significantly improved the other five functions (Wilcoxon signed-rank test, *p* < 0.05), with the greatest improvement in visual function (31%) and communication function (78%).

**Table 3:** Clinical variables comparisons between improvement and unimprovement.

**Variables**	**Improvement (*n* = 15)**	**Unimprovement (*n* = 16)**	**Statistic value**	***P***
**Gender**			NA^a^	0.172
Male	14	11		
Female	1	5		
**Age**	48 (31.0, 61.0)	51 (36, 57.5)	113.5^b^	0.8
**[M(P**_**25**_ **,P**_**75**_ **)]**				
**Etiology**			1.588^c^	0.208
TBI	7	4		
NTBI	8	12		
**Post-injure [M (P**_**25**_ **,P**_**75**_ **), months]**	5 (4.0, 8.0)	7.5 (4.5, 12.0)	217.5 ^b^	0.379
**CRS-R onset [Mean (min, max)]**	8.80 (4.0, 15.0)	8.25 (3.0, 15.0)	86 ^b^	0.188
**Frequencies**			0.819 ^c^	0.479
5 Hz	6	9		
70 Hz	9	7		
**Diagnosis**			11.335^a^	0.002*
VS/UWS	2	8		
MCS–	12	3		
MCS+	1	5		

^a^Fisher exact test; ^b^Mann-Whitney U test; ^c^Chi-square test; *p < 0.05; Etiology (TBI, traumatic brain injuries; NTBI, not traumatic brain injuries); Clinical diagnosis (VS/UWS, Vegetative state or Unresponsive Wakefulness Syndrome; MCS−, minimally conscious state minus; MCS+, minimally conscious state plus; EMCS, emerged from MCS).

In addition, although the effective rate of the 70 Hz was higher than the 5 Hz [56% (9/16) vs. 40% (6/15)], there was no statistically significant difference in the frequency between the effective and ineffective groups (X^2^ = 0.366, *p* = 0.479) (Table 3). Similarly, although the median duration of disease (5 vs. 7.5 months) and age (48 vs. 51 years) were lower in the effective group than in the ineffective group, there was no statistically significant difference between the two groups (*p* > 0.05) (Table 3).

### Regulation characteristics of different frequencies of stSCS

**Figure 8:**
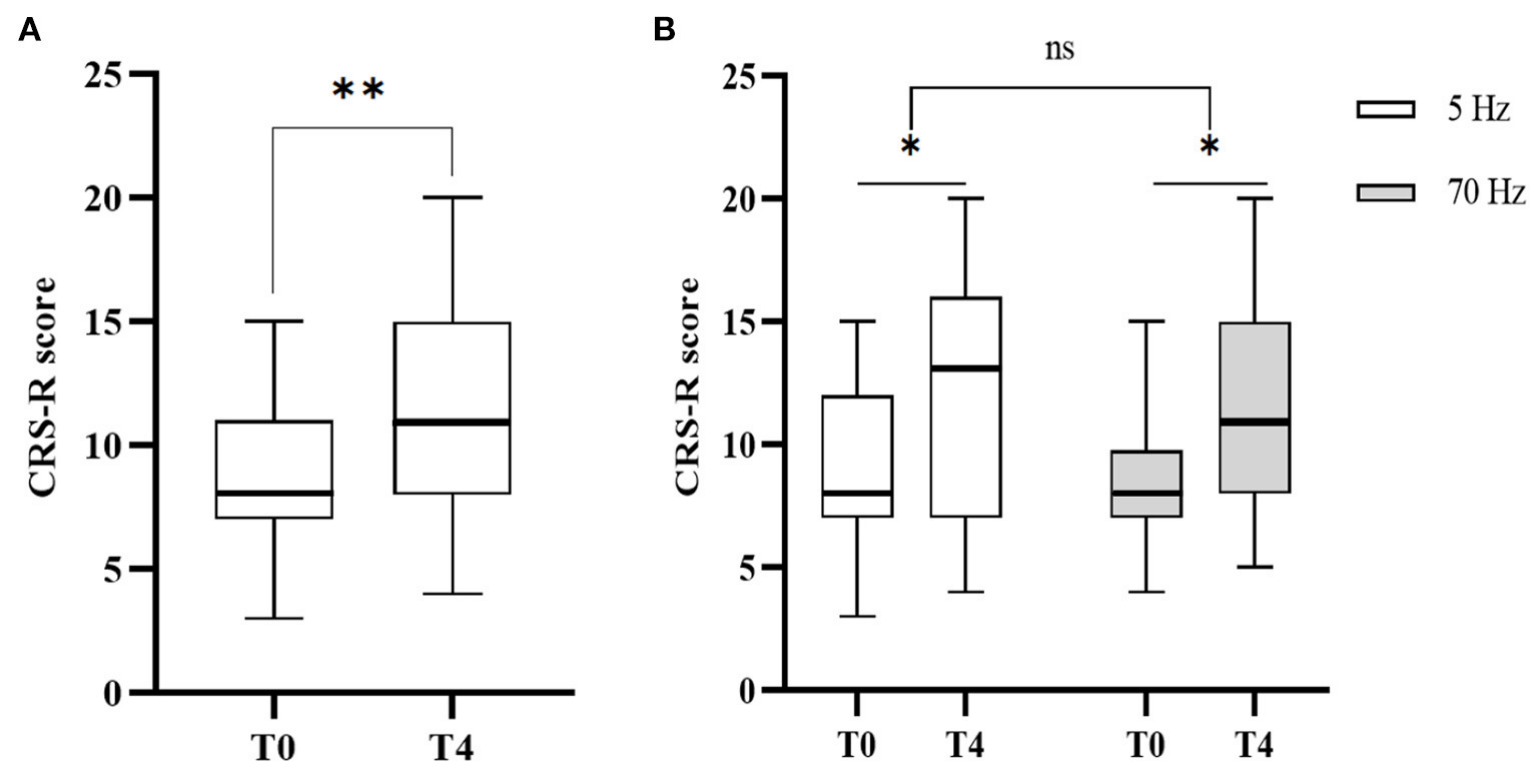
CRS-R changes before the treatment (T0) and 3 months follow up (T4). **(A)** CRS-R changes before and after the treatment. Wilcoxon signed-rank test indicates significant differences (**p < 0.01). **(B)** CRS-R changes in different frequency groups before and after the treatment. Two-way RMANOVA indicates significant differences (*p < 0.05), ns not statistically significant.

st-SCS significantly improved CRS-R scores (T0:8.00 vs. T1:11.00, Z = −3.668, *p* < 0.001) (Figure 8A). One-way RMANOVA revealed a statistically significant main effect of Time (T0, T1, T2, T3, T4) [F_(2.005, 60.163)_ = 15.210, *p* < 0.001)]. *Post hoc* revealed that the CRS-R score failed to improve significantly at 1 week of treatment (T0: 8.52 ± 3.054 vs. T1: 9.19 ± 3.66, *p* = 0.103), while a significant increase in CRS-R score could be seen at 2 weeks of treatment (T1: 9.19 ± 3.66 vs. T2: 10.16 ± 4.19, *p* = 0.001) and a further significant increase in CRS-R score at 1 week after treatment (T2: 10.16 ± 4.188 vs. T3: 11.58 ± 4.82, *p* = 0.004), but the CRS-R score stabilized at 3 months of follow-up without a further increase (T3: 11.58 ± 4.82 vs. T4: 11.68 ± 4.78, *p* = 0.742) (Figure 5B).

As for the frequency subgroup (Figure 8B), regarding the time (T0, T4) * frequency (5 Hz, 70 Hz) RMANOVA suggested a statistically significant effect of time [(*p* = 0.002), *F*_(1, 14)_ = 13.6]. However, there was no statistical difference in the effect of frequency [(*p* = 0.979), *F*_(1, 14)_ = 0.001]. One-way RMANOVA showed statistically significant effects of different frequencies respective time factors (T0, T1, T2, T3, T4) [5 Hz: *F*_(2.017, 28.243)_ = 5.623, *p* = 0.009 < 0.05, 70 Hz: *F*_(1.212, 18.183)_ = 12.438, *p* = 0.002]. *Post hoc* showed different clinical modulation characteristics between 5 and 70 Hz (Figure 5C). 5 Hz showed a significant increase in CRS-R score mainly at 2 weeks of treatment (T1: 10.07 ± 4.367 vs. T2:11.13 ± 5.167, *p* = 0.027), while 1 week after treatment (T2: 11.13 ± 5.167 vs. T3: 11.27 ± 5.133, *p* = 0.334), and 3 months follow-up (T2: 11.13 ± 5.167 vs. T4:11.67 ± 5.233, *p* = 0.486) did not continue to increase. In contrast, 70 Hz was able to significantly increase CRS-R score at 2 weeks of treatment (T1: 8.37 ± 2.754 vs. T2:9.25 ± 2.887, *p* = 0.025) and CRS-R scores continued to significantly increase after 1 week of treatment (T2: 9.25 ± 2.887 vs. T3: 11.88 ± 4.66, *p* = 0.004), but CRS-R scores stabilized during 3 months of follow-up (T3: 11.88 ± 4.66 vs. T4:11.69 ± 4.48, *p* = 0.383).

## Discussion

Our work demonstrates the safety and feasibility of st-SCS in the treatment of pDOC, with an overall effective rate of 48%. At the same time, we found that although there was no difference in the effective rate of st-SCS between 5 and 70 Hz, there were different clinical modulation characteristics, and especially 70 Hz showed a significant delayed effect.

To verify the effectiveness of st-SCS and exclude the interference of natural recovery as much as possible, the study chose the same time of enrollment (3 months after onset brain injury) as conventional SCS (6, 15). And, we adopted a self-controlled design. The stable level of consciousness in pre-treatment and post-treatment may attribute the improvement of CRS-R to the treatment of st-SCS.

It is now generally accepted that VS/UWS and MCS have significant structural differences, with autopsies of VS/UWS patients revealing extensive death of neurons throughout the thalamus, subcortical white matter leading to widespread disconnections between different cortical regions (14), which makes the functional brain regions unable to work together, and information cannot be efficiently integrated and processed. Hence, This Cortico-cortical connectivity in VS/UWS is less likely to enhance through ascending impulses by SCS to reproduce the consciousness network. In contrast, MCS has relatively more intact brain structures, higher plasticity, and higher sensitivity to external stimuli. A series of studies also confirmed that there is a higher therapeutic value of SCS among MCS patietns compared to VS/UWS (6, 13). However, the study found no significant difference in effective rate of st-SCS between MCS and VS/UWS, and we found similar effective rate of st-SCS between MCS+ and VS/UWS with *p* values close to 1. We further subdivided MCS into MCS– and MCS+. The analysis revealed there is significant higher effective rate of st-SCS among MCS patients compared with the VS/UWS and the MCS+. Unlike previous SCS studies, st-SCS was not effective for the MCS+. The findings suggest that st-SCS is difficult to enable pDOC to break through the MCS+ and recover full consciousness.

Patients with emerging from MCS (EMCS) have higher cognitive functions and motor coordination. Both the global neuronal workspace theory (24) and the integrated information theory (25), suggest that consciousness arises from the interaction and integration of information by different neural networks or cognitive modules. The thalamocortical and cortical-cortical connections of the brain network are the core neural loops for the generation and maintenance of consciousness. The frontoparietal cortical network is considered to be the “hub” network of consciousness and is connected *via* the central thalamus. Recent anesthetized macaques studies have found that 50 Hz stimulation of the central thalamus can promote its project to frontoparietal cortex and further strengthens the interconnections between the frontoparietal cortex (26). In the study of the mechanisms of down-up modulation of cortico-cortical connectivity by SCS, our team found significant changes in connectivity within the frontal cortex and across frontal-parietal and frontal-occipital brain regions during SCS stimulation, but only stimulation effects in the frontal cortex remained after cessation of stimulation, while stimulation effects across brain regions returned to pre-stimulation baseline levels (27). Another study also found that only an increase in frontal EEG complexity after SCS stimulation was associated with higher levels of consciousness in pDOC. This shows that the frontal cortex plays a central role in SCS for the regulation of brain activity. We hypothesize that SCS give priority to increasing the level of frontal cortex activity and then recreates the consciousness network through its strengthening of frontal-parietal and frontal-occipital cortical connections. In conclusion, st-SCS may cause an initial restoration of brain regions and connectivity in the consciousness loop by enhancing frontal cortical activity, leading to a substantial improvement in consciousness in MCS– with low levels of consciousness, but consciousness improvement caused by st-SCS stops at MCS+ probably because short-term stimulation does not sufficiently activate the frontoparietal functional network to cause effective connectivity of multiple cognitive modules and prolonged neural remodeling. Our study also found that the delayed effect lasted only 1 week, which corroborates this idea.

The stimulation frequency is one of the most critical parameters for SCS treatment. low-frequencies SCS activate neurons, while high frequencies (>60 Hz) produce inhibitory effects in the field of treatment for pain (28). However, positive recovery of consciousness effects of low-frequency and high-frequency SCS both have been reported in the field of treatment for DOC (6, 12, 13, 15, 29). However, there is no direct clinical study for comparison differences in treatment between low and high-frequency SCS for pDOC. In this regard, this study presents the first EEG-based preferential treatment frequency and compares the difference between 5 and 70 Hz modulation. However, We did not directly find a significant difference of effective rate of st-SCS, which may be limited by the sample size. In addition, we found an additional delayed effect of 70 Hz. In a previous study, functional near-infrared spectroscopy studies found significant increases in hemodynamic responses after a single high-frequency SCS. Especially, significant enhancement of functional connectivity between frontal-occipital lobes occurred after 70 Hz modulation. But no significant post-stimulation effects were found with low-frequency stimulation (30). Our team further found that there was a significant post-effect of 70 Hz SCS based on EEG which showed a significant decrease in path length and a significant increase in small-world effect and tended to the normal control, as well as a strengthening of connectivity between frontal and posterior brain region (31). In conclusion, the sustained improvement of consciousness after high-frequency long-term stimulation may be a result of enhanced neuronal plasticity, allowing for the gradual enhancement of functional connectivity and information interaction in the thalamus-frontal nerve loop, which is closely related to conscious activity, and the recovery of sustained remodeling of functional networks throughout the brain.

The results should be interpreted with caution. Firstly, the study is an exploratory small sample study, and spontaneous recovery could not be completely excluded. Furthermore, small sample study has weak comparability between groups due to the great heterogeneity of pathological damage among individuals of pDOC, which preventing the set of controls group in this study. The relationship between duration of disease and the efficacy was not found in this study. On the one hand, the sample size was insufficient. On the other hand, restricted inclusion for early patients due to the consideration of mitigating the effect of spontaneous recovery on the outcome, which made the overall duration of disease in this study large. Therefore, there is a need to conduct future studies on ultra-early pDOC whose duration of disease is < 3 months. In addition, in this study, we only used the CRS-R to quantify the efficacy of st-SCS, and future studies using more objective neuroimaging and neurophysiological assessment techniques to further understand the mechanisms of neuromodulation. Finally, our work indicates that st-SCS has limited efficacy. The combined activation of multiple brain regions by non-invasive neuromodulation techniques and st-SCS is also a promising therapy for the future.

## Conclusions

In this study, we found for the first time that st-SCS is a safe and effective therapy for patients with pDOC, and it is particularly suitable for MCS–. In addition, we found the modulation characteristics of the two types of frequencies 5 Hz and 70 Hz differed, with the former improving consciousness mainly during stimulation and the latter showing additional post-stimulation delay effects. Although we did not find a significant effect of age and duration of disease on the efficacy of st-SCS, we found that the two factors in the effective group were lower than those in the ineffective group, which may need to be verified with a larger sample size, especially with the inclusion of pDOC patients with duration of diseases < 3 months. In conclusion, this study provides a new perspective on the treatment of pDOC patients with SCS and provides a basis for the selection and modulation of postoperative stimulation parameters.

## Data availability statement

The raw data supporting the conclusions of this article will be made available by the authors, without undue reservation.

## Ethics statement

The study protocol was registered at the Chinese Clinical Trial Registry (ChiCTR2200061278) and was approved by the Ethics Committee of Beijing Tiantan Hospital, Capital Medical University (No. KYSQ 2021-396-01). The verbal and written informed consents were obtained prior to the study. The patients/participants provided their written informed consent to participate in this study.

## Author contributions

Conceptualization: YZ and JH. Methodology: YZ and YY. Data curation: XC and XG. Formal analysis, investigation, and writing—original draft preparation: YZ. Writing—review and editing: JH and JZ. Funding acquisition: JH and LX. Resources: XC. Supervision: JH. All authors contributed to the article and approved the submitted version.

## Funding

This work was supported by the National Natural Science Foundation of China (82272118), National Defense Science and Technology Innovation Special Zone Project of China (17-163-12-ZT-006-003-08&18-H863-02-ZT-008-022-02), Guangdong Provincial Key R&D Programme (2018B030339001), and the Science and Technology Innovation 2030—Brain Science and Brain-like Research Major Project (2021ZD0204203).

## Conflict of interest

The authors declare that the research was conducted in the absence of any commercial or financial relationships that could be construed as a potential conflict of interest.

## Publisher's note

All claims expressed in this article are solely those of the authors and do not necessarily represent those of their affiliated organizations, or those of the publisher, the editors and the reviewers. Any product that may be evaluated in this article, or claim that may be made by its manufacturer, is not guaranteed or endorsed by the publisher.

## Abbreviations

pDOC: Prolonged disorders of consciousness
tDCS: transcranial direct current stimulation
rTMS: repetitive transcranial magnetic stimulation
DBS: Deep Brain Stimulation
SCS: spinal cord stimulation
VNS: vagus nerve stimulation
RMANOVA: repeated measures ANOVA.

## References

[1] Giacino JT Kalmar K Whyte J. The JFK coma recovery scale-revised: measurement characteristics and diagnostic utility. Arch Phys Med Rehabil. (2004) 85:2020–9. 10.1016/j.apmr.2004.02.033 15605342

[2] Bruno MA Vanhaudenhuyse A Thibaut A Moonen G Laureys S. From unresponsive wakefulness to minimally conscious PLUS and functional locked-in syndromes: recent advances in our understanding of disorders of consciousness. J Neurol. (2011) 258:1373–84. 10.1007/s00415-011-6114-x 21674197

[3] Thibaut A Schiff N Giacino J Laureys S Gosseries O. Therapeutic interventions in patients with prolonged disorders of consciousness. Lancet Neurol. (2019) 18:600–14. 10.1016/s1474-4422(19)30031-6 31003899

[4] Edlow BL Claassen J Schiff ND Greer DM. Recovery from disorders of consciousness: mechanisms, prognosis and emerging therapies. Nat Rev Neurol. (2021) 17:135–56. 10.1038/s41582-020-00428-x 33318675 PMC7734616

[5] Schiff ND Giacino JT Kalmar K Victor JD Baker K Gerber M. Behavioural improvements with thalamic stimulation after severe traumatic brain injury. Nature. (2007) 448:600–3. 10.1038/nature06041 17671503

[6] Yang Y He Q Xia X Dang Y Chen X He J. Long-term functional prognosis and related factors of spinal cord stimulation in patients with disorders of consciousness. CNS Neurosci Ther. (2022) 00:1–10. 10.1111/cns.13870 35619213 PMC9253730

[7] Corazzol M Lio G Lefevre A Deiana G Tell L André-Obadia N. Restoring consciousness with vagus nerve stimulation. Curr Biol. (2017) 27:R994–r6. 10.1016/j.cub.2017.07.060 28950091

[8] Schiff ND. Central thalamic deep brain stimulation to support anterior forebrain mesocircuit function in the severely injured brain. J Neural Transm (Vienna). (2016) 123:797–806. 10.1007/s00702-016-1547-0 27113938

[9] Magrassi L Maggioni G Pistarini C Di Perri C Bastianello S Zippo AG. Results of a prospective study (CATS) on the effects of thalamic stimulation in minimally conscious and vegetative state patients. J Neurosurg. (2016) 125:972–81. 10.3171/2015.7.Jns15700 26745476

[10] Chudy D Deletis V Almahariq F Marčinković P Škrlin J Paradžik V. Deep brain stimulation for the early treatment of the minimally conscious state and vegetative state: experience in 14 patients. J Neurosurg. (2018) 128:1189–98. 10.3171/2016.10.Jns161071 28621620

[11] Kanno T Kamel Y Yokoyama T Shoda M Tanji H Nomura M. Effects of dorsal column spinal cord stimulation (DCS) on reversibility of neuronal function–experience of treatment for vegetative states. Pacing Clin Electrophysiol. (1989) 12:733–8. 10.1111/j.1540-8159.1989.tb02724.x 2470059

[12] Kanno T Morita I Yamaguchi S Yokoyama T Kamei Y Anil SM. Dorsal column stimulation in persistent vegetative state. Neuromodulation. (2009) 12:33–8. 10.1111/j.1525-1403.2009.00185.x 22151220

[13] Yamamoto T Watanabe M Obuchi T Kobayashi K Oshima H Fukaya C. Spinal cord stimulation for vegetative state and minimally conscious state: changes in consciousness level and motor function. Acta Neurochir Suppl. (2017) 124:37–42. 10.1007/978-3-319-39546-3_6 28120050

[14] Della Pepa GM Fukaya C La Rocca G Zhong J Visocchi M. Neuromodulation of vegetative state through spinal cord stimulation: where are we now and where are we going? Stereotact Funct Neurosurg. (2013) 91:275–87. 10.1159/000348271 23797266

[15] Xu Y Li P Zhang S Wang Y Zhao X Wang X. Cervical spinal cord stimulation for the vegetative state: a preliminary result of 12 cases. Neuromodulation. (2019) 22:347–54. 10.1111/ner.12903 30548939

[16] Zhao L Song T. Case report: Short-term spinal cord stimulation and peripheral nerve stimulation for the treatment of trigeminal postherpetic neuralgia in elderly patients. Front Neurol. (2021) 12:713366. 10.3389/fneur.2021.713366 34413827 PMC8368125

[17] Wan CF Song T. Efficacy of pulsed radiofrequency or short-term spinal cord stimulation for acute/subacute zoster-related pain: a randomized, double-blinded, controlled trial. Pain Phys. (2021) 24:215–22. 33988940

[18] Deer TR Mekhail N Provenzano D Pope J Krames E Leong M. The appropriate use of neurostimulation of the spinal cord and peripheral nervous system for the treatment of chronic pain and ischemic diseases: the Neuromodulation Appropriateness Consensus Committee. Neuromodulation. (2014) 17:515–50; discussion 50. 10.1111/ner.12208 25112889

[19] Sun W Jin Y Liu H Yang D Sun T Wang Y. Short-term spinal cord stimulation is an effective therapeutic approach for herpetic-related neuralgia—a Chinese Nationwide Expert Consensus. Front Aging Neurosci. (2022) 14:939432. 10.3389/fnagi.2022.939432 36204548 PMC9530637

[20] Wutzl B Golaszewski SM Leibnitz K Langthaler PB Kunz AB Leis S. Narrative review: quantitative EEG in disorders of consciousness. Brain Sci. (2021) 11:697. 10.3390/brainsci11060697 34070647 PMC8228474

[21] Bai Y Lin Y Ziemann U. Managing disorders of consciousness: the role of electroencephalography. J Neurol. (2021) 268:4033–65. 10.1007/s00415-020-10095-z 32915309 PMC8505374

[22] Bai Y Xia X Li X Wang Y Yang Y Liu Y. Spinal cord stimulation modulates frontal delta and gamma in patients of minimally consciousness state. Neuroscience. (2017) 346:247–54. 10.1016/j.neuroscience.2017.01.036 28147246

[23] Wang Y Bai Y Xia X Yang Y He J Li X. Spinal cord stimulation modulates complexity of neural activities in patients with disorders of consciousness. Int J Neurosci. (2020) 130:662–70. 10.1080/00207454.2019.1702543 31847650

[24] Dehaene S Sergent C Changeux JP A. neuronal network model linking subjective reports and objective physiological data during conscious perception. Proc Natl Acad Sci USA. (2003) 100:8520–5. 10.1073/pnas.1332574100 12829797 PMC166261

[25] Tononi G. Consciousness as integrated information: a provisional manifesto. Biol Bull. (2008) 215:216–42. 10.2307/25470707 19098144

[26] Redinbaugh MJ Phillips JM Kambi NA Mohanta S Andryk S Dooley GL. Thalamus modulates consciousness via layer-specific control of cortex. Neuron. (2020) 106:66–75.e12. 10.1016/j.neuron.2020.01.005 32053769 PMC7243351

[27] Bai Y Xia X Liang Z Wang Y Yang Y He J. Frontal connectivity in EEG gamma (30-45 Hz) respond to spinal cord stimulation in minimally conscious state patients. Front Cell Neurosci. (2017) 11:177. 10.3389/fncel.2017.00177 28701924 PMC5487422

[28] Yampolsky C Hem S Bendersky D. Dorsal column stimulator applications. Surg Neurol Int. (2012) 3:S275–89. 10.4103/2152-7806.103019 23230533 PMC3514915

[29] Xia X Yang Y Guo Y Bai Y Dang Y Xu R. Current status of neuromodulatory therapies for disorders of consciousness. Neurosci Bull. (2018) 34:615–25. 10.1007/s12264-018-0244-4 29916112 PMC6060218

[30] Si J Dang Y Zhang Y Li Y Zhang W Yang Y. Spinal cord stimulation frequency influences the hemodynamic response in patients with disorders of consciousness. Neurosci Bull. (2018) 34:659–67. 10.1007/s12264-018-0252-4 29995275 PMC6060214

[31] Liang Z Na RE Yong WA Jiani LI Yang BA Xiaoli LI. study of the brain function evaluation in the minimally conscious state using cross-sample entropy based on brain network measure under the spinal cord stimulation (in Chinese). Sci Sin Inform. (2021) 51:19. 10.1360/SSI-2020-0010

